# A Rare Case of Dupilumab Monotherapy in Pemphigoid Nodularis and a Comprehensive Literature Review

**DOI:** 10.7759/cureus.99280

**Published:** 2025-12-15

**Authors:** Salman M Albthali, Falah M Alajmi, Saood Almutairi, Humoud Al-sabah

**Affiliations:** 1 Dermatology, As'ad Al-Hamad Dermatology Center, Sulaibikhat, KWT; 2 Dermatology, Abdulkareem Al-Saeed Dermatology Center, Kuwait City, KWT

**Keywords:** biologics, bullous pemphigoid, case report, dupilumab, il-13, il-4, monotherapy, pemphigoid nodularis, steroid-sparing

## Abstract

Pemphigoid nodularis (PN) is a rare, refractory variant of bullous pemphigoid (BP) characterized by prurigo nodularis-like lesions and the immunopathologic features of BP. Its management is challenging, especially in elderly patients for whom conventional immunosuppressants pose significant risks. Dupilumab, a monoclonal antibody inhibiting IL-4 and IL-13 signaling, has emerged as a promising therapy for BP, but its efficacy in PN is not well-established. A 71-year-old female patient presented with a 10-day history of intensely pruritic, tense blisters and nodules on her hands, feet, and trunk. Histopathology confirmed acanthosis and hyperkeratosis of epidermis, also subepidermal blister with eosinophils, and direct immunofluorescence revealed linear IgG and C3 deposition at the basement membrane zone, diagnosing PN. Due to her age and contraindications for long-term steroids, she was started on dupilumab monotherapy (600 mg loading dose, then 300 mg every two weeks). The patient experienced rapid pruritus relief within 72 hours and significant lesion healing by week 4, with no adverse events. This case demonstrates that dupilumab monotherapy is a highly effective and well-tolerated treatment for PN. It provides rapid, steroid-free disease control, supporting its use as a first-line biologic agent for this debilitating condition. This report is bolstered by a growing body of evidence from real-world studies on dupilumab in BP.

## Introduction

Pemphigoid nodularis (PN) is a rare autoimmune blistering disease first delineated by Yashimoto et al. in 1984 [1]. It is clinically characterized by a dual morphology: prurigo nodularis-like hyperkeratotic, excoriated papules and nodules, and the classic tense blisters of bullous pemphigoid (BP) [2,3]. This creates a significant diagnostic challenge, often leading to delays in correct treatment. Immunopathologically, PN is indistinguishable from BP, featuring IgG and/or C3 autoantibodies targeting the hemidesmosomal proteins BP180 (type XVII collagen) and BP230, leading to subepidermal blister formation [4,5].

The pathogenesis of PN, like BP, is driven by a T helper 2 (Th2)-polarized immune response. This involves elevated levels of cytokines IL-4, IL-5, IL-13, and IL-31, which promote B-cell activation, eosinophil recruitment, mast cell degranulation, and the profound pruritus that is a hallmark of the disease [6,7]. The role of IL-4 and IL-13 is particularly crucial, as they directly contribute to IgE class switching, eosinophil infiltration, and skin barrier dysfunction [8].

Managing PN is notoriously difficult. The disease is often chronic and refractory to first-line therapies [9]. Patients are typically elderly and possess multiple comorbidities, making them exceptionally vulnerable to the severe adverse effects of high-potency topical and systemic corticosteroids (CS), which remain the mainstay of initial treatment [10,11]. Conventional steroid-sparing agents (e.g., azathioprine, mycophenolate mofetil, methotrexate) also carry risks of hepatotoxicity, myelosuppression, and increased susceptibility to infections, necessitating frequent monitoring [12]. This high iatrogenic risk profile has spurred the search for targeted, safer therapeutic alternatives.

Dupilumab, a fully human monoclonal antibody that binds to the IL-4 receptor alpha subunit, antagonizes the signaling of both IL-4 and IL-13. It is approved for atopic dermatitis, asthma, and chronic rhinosinusitis with nasal polyps [13]. By blocking these key Th2 cytokines, dupilumab directly targets the central inflammatory pathway in BP and PN, reducing IgE levels, eosinophil activity, and pruritus [14]. Recent large-scale real-world studies have robustly demonstrated its efficacy and safety in BP [15,16]. However, evidence for its use specifically in PN remains limited to sparse case reports [17,18]. We present a case of PN successfully treated with dupilumab monotherapy and provide a comprehensive review of the current literature on innovative treatments for autoimmune blistering diseases.

## Case presentation

A 71-year-old female patient presented with "itchy, tense blisters on her hands, feet, and trunk" for 10 days. The patient developed abruptly occurring, intensely pruritic, tense blisters on the dorsal aspects of her hands, feet, and trunk approximately 10 days prior to presentation. The lesions were accompanied by a profound sensation of itch. There was no prior history of similar episodes or a known diagnosis of an autoimmune blistering disease. The patient is a known case of multiple comorbidities, including hypertension, diabetes mellitus, and osteoarthritis, and has no history of malignancy. The patient is on lisinopril, gliclazide, and occasional ibuprofen for arthritic pain. No new medications were started in the six months preceding the eruption. Physical examination revealed multiple, firm, tense vesicles and bullae on an erythematous base distributed over the dorsal hands, feet, and trunk. Several older lesions appeared as eroded, crusted papules and nodules, consistent with the prurigo-like morphology of PN. Nikolsky's sign was negative. Mucous membranes were spared.

Multiple investigations were conducted as part of diagnoses. Skin biopsy was taken from two sites: the first was from the blister site, which showed a subepidermal blister with a prominent inflammatory infiltrate rich in eosinophils, and the second biopsy was from the nodular site, which showed acanthosis and hyperkeratosis of the epidermis. Direct, indirect, and enzyme-linked immunosorbent assay (ELISA) serologies were requested as part of the full investigation to confirm the diagnoses. Direct immunofluorescence (DIF) from the perilesional skin biopsy revealed linear deposition of IgG and C3 along the basement membrane zone, confirming the diagnosis of an autoimmune bullous disease consistent with pemphigoid. Indirect immunofluorescence (IIF) salt-split revealed linear deposition of IgG at the epidermal side. ELISA serology and anti-BP180 NC16A antibody titers were significantly elevated at 128 U/mL (normal <9 U/mL). Anti-BP230 antibodies were also positive; all the results led us to confirm our diagnosis of PN.

**Figure 1:**
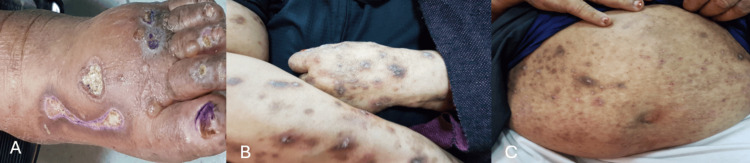
Clinic features of pemphigoid nodularis (A-C) revealed multiple, firm, tense vesicles and bullae on an erythematous base distributed over the dorsal hands, feet and trunk. Several older lesions appeared as eroded, crusted papules and nodules, consistent with the prurigo-like morphology of pemphigoid nodularis

Clinically, PN is characterized by the presence of intensely pruritic (itchy) nodules that resemble those of prurigo nodularis. These nodules are often excoriated due to chronic scratching and are typically located on the extremities and trunk. A distinguishing feature is the occasional presence of blisters (bullae) or vesicles, which can appear on top of the nodules or on seemingly normal skin. The bullous lesions may be present at the initial presentation or develop later in the disease course (Figures 1A-1C).

**Figure 2:**
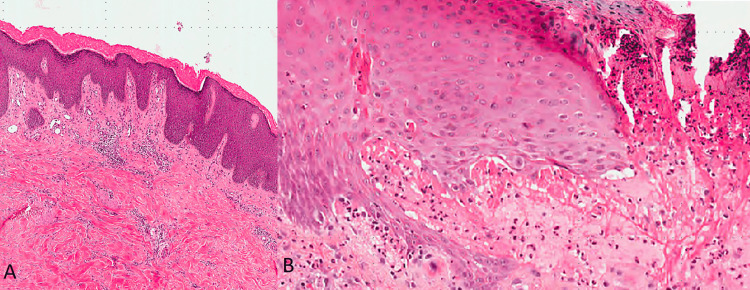
Histopathology of pemphigoid nodularis (H&E) H&E: hematoxylin and eosin (A) A biopsy of the nodular lesion shows acanthosis and hyperkeratosis of epidermis. (B) A biopsy of the blister site shows subepidermal vesicles

A biopsy of a nodular lesion in PN often shows nonspecific findings similar to prurigo nodularis. The epidermal biopsy revealed marked hyperkeratosis (thickening of the outer layer of the skin) and acanthosis (thickening of the epidermis) in the epidermal (Figure 2A). The dermal biopsy showed a mixed inflammatory infiltrate in the dermis, composed of lymphocytes, plasma cells, neutrophils, and a prominent number of eosinophils. This eosinophil-rich infiltrate can be a clue to an underlying BP process. In some cases, a subepidermal blister may be seen (Figure 2B).

**Figure 3:**
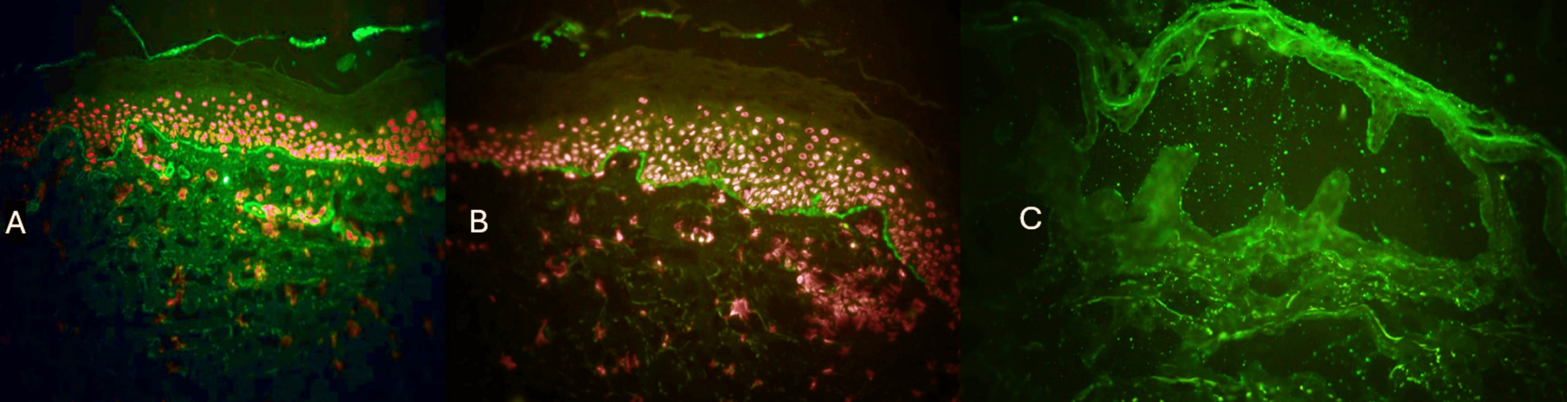
Direct and indirect immunofluorescence (A and B) Direct immunofluorescence revealed linear deposition of IgG and C3, respectively. (C) Salt-split indirect immunofluorescence revealed linear deposition of IgG at the epidermal side

Immunofluorescence is the gold standard for diagnosing PN and differentiating it from other pruritic nodular conditions. This finding is identical to that seen in classic BP and is the definitive feature that distinguishes PN from prurigo nodularis. A direct immunofluorescence was performed on a perilesional skin biopsy (skin near a lesion). It shows a characteristic linear deposition of IgG and/or C3 along the dermal-epidermal junction (basement membrane zone). This pattern is identical to classic BP and confirms the autoimmune nature of the disease (Figures 3A-3B). A salt-split indirect immunofluorescence revealed linear deposition of IgG at the epidermal side (Figure 3C). While the clinical and histopathological findings of PN may overlap significantly with benign conditions like prurigo nodularis, the definitive diagnosis is established by the characteristic linear pattern of IgG and C3 deposition at the dermal-epidermal junction revealed by direct immunofluorescence.

Treatment and course

**Figure 4:**
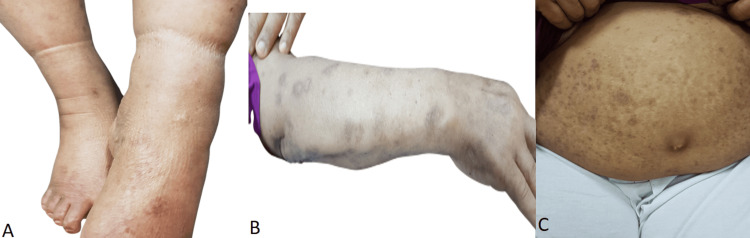
Post-dupilumab treatment images (A-C) shows a complete healing from pemphigoid nodularis and also shows post-inflammatory hyperpigmentation without any nodules or blisters. These pictures were taken six months after initiating dupilumab

Given the patient's diagnosis and the desire to avoid the systemic side effects of corticosteroids, a decision was made to initiate dupilumab monotherapy. The patient received a loading dose of 600 mg subcutaneously, followed by 300 mg every two weeks as a maintenance dose for three months. Topical clobetasol propionate 0.05% ointment was applied twice daily to active lesions for the first two weeks only. The patient reported a dramatic reduction in pruritus within 72 hours of the first dose. Existing lesions began to heal progressively, with complete re-epithelialization observed at the week 4 follow-up. The topical steroid was discontinued after week 2. The patient tolerated the treatment exceptionally well with no reported adverse events. A plan was made to continue dupilumab for at least six months and monitor clinical response and BP180 titers. After three months of starting dupilumab, the disease was well-controlled with the absence of any new lesions and marked improvement of itching. All old lesions improved, leaving only post-inflammatory hyperpigmentation, and itching improved completely. The frequency of dupilumab injections was reduced to once every four weeks for three months, then once every six weeks (Figures 4A-4C).

The patient started to relapse with a few new nodules while on treatment, once every six weeks. The frequency of injections increased again to 300 mg every two weeks, and a topical potent steroid was applied to active lesions for two weeks. Lesion and itching were well-controlled again after one month. Currently, she is maintained on dupilumab 300 mg every four weeks. No side effects related to dupilumab have been observed to date.

## Discussion

Comprehensive literature review and discussion

The management of autoimmune blistering diseases, particularly in elderly populations, is pivoting toward targeted biologic therapies to mitigate the high burden of morbidity from traditional immunosuppressants. Our case of successful dupilumab monotherapy in PN contributes to this evolving paradigm and is supported by a robust foundation of evidence. The efficacy of dupilumab in BP and PN is underpinned by its precise mechanism of action, which directly counters the Th2-dominant immunopathology. IL-4 and IL-13 are pivotal cytokines in this pathway: they promote B-cell activation and autoantibody production (including IgE), recruit and activate eosinophils, and contribute to impaired skin barrier function [6,8,19]. By blocking the shared IL-4α receptor, dupilumab effectively dampens this entire cascade. This explains the rapid improvement in pruritus (often within days) observed in our patient and others [20], as IL-4 and IL-13 are key drivers of itch signaling. Furthermore, the swift cessation of blistering aligns with the drug's effect on reducing inflammatory cell recruitment and preventing their localization to the dermal-epidermal junction.

Our findings are consistent with large-scale real-world studies. A multicenter retrospective cohort study in China of 134 patients with moderate-to-severe BP found that dupilumab (600 mg/300 mg regimen) led to rapid disease control, with a significant proportion of patients achieving complete remission by week 12. It provided pronounced itch relief and a substantial steroid-sparing effect, which is critical for older adults with comorbidities [15]. Similarly, the largest European real-world series, an ambispective study across 34 Spanish hospitals involving 103 BP patients, confirmed that dupilumab is an effective, rapid-acting, and safe treatment. The study highlighted that it drastically reduced the need for systemic corticosteroids and that earlier initiation was associated with superior outcomes [16]. These studies position dupilumab as a cornerstone therapy for BP, a conclusion that can logically be extended to its variant, PN. The standard first-line treatment for BP and PN has long been systemic corticosteroids, despite a one-year mortality rate that can exceed 20% in elderly patients, largely attributable to treatment complications [10,11]. The imperative for steroid-sparing strategies is clear.

Rituximab

An anti-CD20 monoclonal antibody is highly effective for refractory BP [21]. The Ritux 3 trial demonstrated its superiority over corticosteroids alone for achieving sustained remission [22]. However, its use is associated with a risk of severe infections, especially in the very elderly, and requires intravenous infusion [23].

Omalizumab

An anti-IgE antibody has shown efficacy in case series, particularly in patients with elevated IgE levels [24,25]. Its role is more niche, and robust controlled trial data are lacking.

Intravenous Immunoglobulin (IVIG)

IVIG is effective for refractory cases but is costly, requires intravenous access, and has limited availability [26]. In this context, dupilumab offers a compelling alternative: high efficacy, a favorable safety profile, subcutaneous administration, and a specific mechanism of action tailored to the disease's pathophysiology. Its use as monotherapy, as demonstrated in our case, is a significant advance, potentially eliminating systemic immunosuppression entirely.

While the evidence for dupilumab in BP is strong, reports on its use in PN are scarce. A few case reports have documented success, mirroring our experience [17,18,27]. Our case strengthens this evidence by demonstrating a rapid and complete response with monotherapy, highlighting its potential as a first-line agent for this specific variant. The similar immunopathogenesis between BP and PN provides a strong rationale for this cross-efficacy.

## Conclusions

This case report provides strong evidence that dupilumab monotherapy is a transformative treatment option for PN. It offers rapid, steroid-free disease control with an excellent safety profile, making it ideally suited for the elderly, multimorbid patient population typically affected by this disease. While randomized controlled trials specifically for PN are unlikely due to its rarity, the accumulating real-world evidence for BP and case reports for PN should encourage the inclusion of dupilumab in treatment guidelines for this challenging disease. Future research should focus on long-term outcomes, optimal treatment duration, and biomarker prediction of response.

## References

[1] Yashimoto T, Otsuka F, Takayama H, Manabe M, Koyama K, Kotake H, Tomii K (1984). A case of bullous pemphigoid with nodular lesions. Nishinihon J Dermatol.

[2] Vornicescu C, Șenilă SC, Cosgarea R, Candrea E, Pop AD, Ungureanu L (2019). Pemphigoid nodularis-rare presentation of bullous pemphigoid: a case report and literature review. Exp Ther Med.

[3] Granados-Betancort E, Sánchez-Díaz M, Muñoz-Barba D, Arias-Santiago S (2014). Omalizumab and dupilumab for the treatment of bullous pemphigoid: a systematic review. J Clin Med.

[4] Holm JG, Agner T, Sand C, Thomsen SF (2020). Dupilumab for prurigo nodularis: case series and review of the literature. Dermatol Ther.

[5] Olbrich H, Sadik CD, Ludwig RJ, Thaci D, Boch K (2023). Dupilumab in inflammatory skin diseases: a systematic review. Biomolecules.

[6] Jiang C, Adjei S, Santiago S, Lu J, Duran M, Tyring S (2023). Novel use of dupilumab in pemphigus vulgaris and pemphigus foliaceus. JAAD Case Reports.

[7] Hsieh CY, Tsai TF (2022). Management of coexisting bullous pemphigoid and psoriasis: a review. Am J Clin Dermatol.

[8] Chen HC, Wang CW, Toh WH, Lee HE, Chung WH, Chen CB (2023). Advancing treatment in bullous pemphigoid: a comprehensive review of novel therapeutic targets and approaches. Clin Rev Allergy Immunol.

[9] Murrell DF, Joly P, Werth VP (2024). Study design of a phase 2/3 randomized controlled trial of dupilumab in adults with bullous pemphigoid: LIBERTY-BP ADEPT. Adv Ther.

[10] Lovegrove FE, Sauder MB, Mufti A (2025). Navigating bullous pemphigoid: consensus recommendations for diagnosis and management-a Canadian perspective. J Cutan Med Surg.

[11] Wang SH, Shan Y, Li SZ, Zuo YG (2023). Anti-interleukin 4 receptor α antibody for the treatment of Chinese bullous pemphigoid patients with diverse comorbidities and a 1-year follow-up: a monocentric real-world study. Front Immunol.

[12] Akbarialiabad H, Schmidt E, Patsatsi A (2025). Bullous pemphigoid. Nat Rev Dis Primers.

[13] Werth VP, Murrell DF, Joly P, Heck R, Orengo JM, Ardeleanu M, Hultsch V (2024). Pathophysiology of bullous pemphigoid: role of type 2 inflammation and emerging treatment strategies (narrative review). Adv Ther.

[14] Lan CC, Chung WH, Chen YY (2025). Taiwanese Dermatological Association consensus for the diagnosis and management of prurigo nodularis. Dermatologica Sinica.

[15] Joly P, Roujeau JC, Benichou J (2002). A comparison of oral and topical corticosteroids in patients with bullous pemphigoid. N Engl J Med.

[16] Joly P, Maho-Vaillant M, Prost-Squarcioni C (2017). First-line rituximab combined with short-term prednisone versus prednisone alone for the treatment of pemphigus (Ritux 3): a prospective, multicentre, parallel-group, open-label randomised trial. Lancet.

[17] Kaye A, Gordon SC, Deverapalli SC, Her MJ, Rosmarin D (2018). Dupilumab for the treatment of recalcitrant bullous pemphigoid. JAMA Dermatol.

[18] Cao P, Xu W, Jiang S, Zhang L (2023). Dupilumab for the treatment of prurigo nodularis: a systematic review. Front Immunol.

[19] Pratasava V, Sahni VN, Suresh A, Huang S, Are A, Hsu S, Motaparthi K (2021). Bullous pemphigoid and other pemphigoid dermatoses. Medicina.

[20] Keow S, Lu B, Xiong G, Yu E, Weng D, Abu-Hilal M (2025). Patient outcomes and safety of combination biologic therapy with dupilumab: a systematic review. Ann Allergy Asthma Immunol.

[21] Patel D, Rosenberg J, Cohen J, Holland KE (2025). Effective treatment of recalcitrant Hailey-Hailey disease with dupilumab. JAAD Case Reports.

[22] Schmidt E, Zillikens D (2013). Pemphigoid diseases. Lancet.

[23] Fässler M, Schlapbach C (2020). Granuloma annulare arising under systemic psoriasis therapy successfully treated with adalimumab. JAAD Case Rep.

[24] Maronese CA, Cassano N, Genovese G, Foti C, Vena GA, Marzano AV (2022). The intriguing links between psoriasis and bullous pemphigoid. J Clin Med.

[25] Zhang J, Wang SH, Zuo YG (2023). Paradoxical phenomena of bullous pemphigoid induced and treated by identical biologics. Frontiers in Immunology.

[26] Thevan J, Schmauch E, Nilsson J (2024). Fast itch relief during dupilumab predicts clinical efficacy in bullous pemphigoid: a retrospective cohort study. Dermatology.

[27] Ilaria P, Nevena S, Ersilia T (2024). Potential indications of dupilumab in Th-2 inflammatory disease. Rev Recent Clin Trials.

